# A Psychopathic Ward at the Philadelphia General Hospital

**Published:** 1914-05

**Authors:** 


A PSYCHOPATHIC WARD AT THE PHILADELPHIA GENERAL HOSPITAL.—The proposed psychopatchic ward for the examination and treatment of suspected cases of insanity was approved by the State Board of Health and Charities April 11th. Persons suspected of mental disease or defects will be sent to this ward. If the patient is found to be insane after a period of thirty days of observation he will be sent to the State Hospital at Norristown. The last Legislature appropriated $10,000 toward the establishment of such a ward, and plans are being made to take temporary care of at least 140 persons. An interesting feature will be the introduction of a system of treatment which will enable many of the patients to return to their homes, under the supervision of physicians and social workers.—*New York Medical Journal.*

